# Clinical Performance and Calibration of the PROFUND Index in Hospitalized and Ambulatory Complex Chronic Patients: A Real-World Retrospective Cohort Study

**DOI:** 10.3390/jcm15114040

**Published:** 2026-05-23

**Authors:** Jorge Martins, Susana Viana, Inês Chora, Fernando Friões

**Affiliations:** 1 Internal Medicine Department, ULS Matosinhos, 4464-513 Senhora da Hora, Portugal; susana.viana@ulsm.min-saude.pt (S.V.); ines.chora@ulsm.min-saude.pt (I.C.); 2 Porto Medical School, University of Porto, 4200-500 Porto, Portugal; fbfrioes@gmail.com; 3 Department of Medicine and Cardiovascular Research and Development Center (UnIC@RISE), ULS São João, 4050-377 Porto, Portugal

**Keywords:** prognosis, case management, multimorbidity

## Abstract

**Background/Objectives:** Complex chronic patients represent a heterogeneous and high-risk population, for whom accurate prognostic tools are essential to guide clinical decision-making, optimize resource allocation, and support tailored interventions. The PROFUND index was developed for mortality prediction in polypathological patients, but its performance has not yet been evaluated in an ambulatory integrated care model. **Methods:** A retrospective observational study was conducted using two cohorts. Cohort H included complex chronic patients admitted to the Internal Medicine Department between March 2023 and February 2024. Cohort A comprised complex chronic patients followed by a multidisciplinary chronic care program between November 2016 and December 2023. PROFUND scores were derived from electronic health records. Discrimination for 12-month mortality was assessed using Kaplan–Meier curves, log-rank tests, and receiver operating characteristic curve analysis. Calibration was evaluated by comparing observed mortality with expected mortality based on the original PROFUND index and improved through intercept and slope recalibration. **Results:** A total of 660 patients were included in cohort H and 540 in cohort A. One-year mortality was 38.0% and 30.2%, respectively. Discriminatory performance was good in hospitalized patients (AUC 0.760; 95% CI 0.724–0.797) and moderate to good in ambulatory patients (AUC 0.705; 95% CI 0.656–0.754). Calibration analyses demonstrated systematic overestimation of mortality, particularly in the ambulatory cohort and intermediate–high risk strata, while recalibration improved agreement between predicted and observed risks. **Conclusions:** The PROFUND index provides useful risk stratification for 12-month mortality in CCP across care settings but overestimates absolute risk, particularly in ambulatory case management populations. Local recalibration may improve prognostic accuracy, support individualized care planning, and advance care planning discussions and allocation of multidisciplinary follow-up intensity.

## 1. Introduction

The growing number of individuals living with multimorbidity worldwide in recent decades [1] and the consequent patient’s loss of functional capacity [2] and quality of life [3], as well as increased use of healthcare services and associated costs [4,5], have driven the search for a model that best responds to the needs of this population. Traditional acute-care approaches are not suitable for these patients, given the biopsychosocial complexity inherent to multimorbidity, in which the patient’s attitude toward illness plays a decisive role in management [6]. Multidisciplinary care, community involvement, case management, and promotion of health literacy have been among the strategies developed to address these patients [7], used individually or in combination, particularly in a subgroup of multimorbid individuals whose social determinants of health significantly affect the management of their chronic conditions. They are referred to as complex chronic patients (CCPs) [8] or polypathological patients. While the concepts substantially overlap, the term “polypathological patients” primarily refers to multimorbidity, whereas “CCP” encompasses a broader framework that emphasizes complexity of care and resource utilization. Although overall results have been positive, they have not been consistent or easily reproducible due to differences across national healthcare systems. CCPs represent a relatively small but disproportionately resource-intensive segment of the population [9] and exhibit marked heterogeneity, with distinct clusters characterized by different clinical and social needs, which may explain the limited effectiveness of uniform interventions and highlight the need for tailored approaches [10]. Patients with predominant psychosocial complexity require an individualized and dynamic social intervention rather than intensive clinical management. Conversely, patients with palliative needs, also considered advanced CCP, require specialized care focused on the specific demands characteristic of this phase [11]. Therefore, practical and easily applicable prognostic tools are needed to support clinical decision-making, care planning, and the allocation of healthcare resources in emerging models of integrated care focused on CCPs.

In Spain, throughout the first decade of the 21st century, several regional strategies were developed for the identification and management of CCPs [12]. Within this context, a predictive model for polypathological patients, known as the PROFUND index, was developed [13] (Table 1). It uses nine independent mortality predictors to calculate the score.

Based on the total score, patients are categorized into four groups according to one-year mortality risk (Table 2).

The discriminatory power of the PROFUND index in a population of polypathological patients was considered good, with an area under the curve (AUC) of 0.734 (95% confidence intervals (CIs) 0.71–0.76) [13]. In the same population, the Charlson Comorbidity Index showed an AUC of 0.594 (95% CI 0.56–0.62). Subsequent studies [14] evaluated the discriminatory performance of the PROFUND index in polypathological patients hospitalized in an internal medicine ward (AUC 0.725; 95% CI 0.670–0.781) and in a geriatrics ward (AUC 0.546; 95% CI 0.448–0.644). Dino Moretti et al. [15] compared the PROFUND index (AUC 0.712; 95% CI 0.607–0.817) with clinicians’ intuition (AUC 0.561; 95% CI 0.458–0.664), and another study [16] tested the ability of the PROFUND index to predict in-hospital mortality (AUC 0.76; 95% CI 0.628–0.891) compared to C-reactive protein, albumin, and red blood cell distribution. Other studies assessed the performance of the PROFUND index in patients with heart failure [17,18], ambulatory polypathological patients [19] and using different follow-up intervals, including the prediction of four-year mortality [20], with consistently reported favorable predictive performance. No study has evaluated the PROFUND index in ambulatory CCPs under case management models. These models may modify short-term prognosis through their impact on early monitoring, the empowerment of patients and caregivers to manage chronic conditions, and prompt intervention during episodes of acute decompensation.

The aim of this study was to evaluate the real-world clinical performance, discrimination, and calibration of the PROFUND index in hospitalized CCPs and ambulatory CCPs integrated in a case management program.

## 2. Methods

In this retrospective observational study, the investigators collected data from two populations of CCPs. The first population, hereafter referred to as cohort H, consists of patients meeting CCP criteria who were admitted to the Internal Medicine Department of an urban hospital and subsequently discharged alive between March 2023 and February 2024. The second population, referred to as cohort A, comprises patients from the same hospital enrolled in a multidisciplinary team dedicated to outpatient follow-up of CCPs, known as the Complex Chronic Patient Support Team (CCPST), between 2 November 2016 and 31 December 2023. The CCPST monitors CCPs through case management, home-based support provided by internal medicine physicians, and the development of an individualized care plan designed to address the clinical and biopsychosocial determinants of complexity.

Patients are classified as CCP if they meet at least three of the following five criteria:

1. Age ≥ 75 years;
2. Five or more emergency department visits within the previous 365 days;
3. Three or more hospital admissions within the previous 365 days;
4. Chronic use of six or more medications;
5. At least three of the following seven chronic conditions: diabetes, active cancer, cerebrovascular disease, heart failure, chronic obstructive pulmonary disease/chronic respiratory failure, chronic kidney disease, or chronic liver disease.

The CCP identification criteria were based on institutional protocols developed for the CCPST and aligned with previously described complex chronic patient identification frameworks.

Exclusion criteria for cohort H included enrolment in the CCPST either during the study period or before the index admission. For cohort A, exclusion criteria at the time of program admission included patients followed by the home-based palliative care team and those who declined to join the CCPST.

For the identification of cohort H, investigators reviewed the daily list of admissions to the Internal Medicine Department and applied inclusion and exclusion criteria through examination of electronic health records (EHRs). Cohort A consisted of all patients enrolled in the CCPST during the predefined period. For all patients, investigators extracted the data required to calculate the PROFUND index from the EHRs, including age, sex, Barthel index, presence of the seven chronic conditions used in the CCP classification system, number of emergency department visits and hospital admissions in the previous year, hemoglobin (Hb) level at admission, caregiver status, New York Heart Association (NYHA) or modified Medical Research Council (mMRC) classification, documentation of delirium during the last hospitalization, and presence of active neoplasia or dementia. Investigators determined the one-year survival status from the date of discharge (cohort H) or CCPST enrolment (cohort A). Using these data, the PROFUND score was calculated, and patients were categorized according to the score-based risk groups. For patients who did not survive up to 12 months, we recorded time to death for survival curve estimation.

Investigators extracting EHR data were not formally blinded to survival outcomes due to the retrospective nature of the study. PROFUND variables were collected using predefined objective criteria based on routinely documented clinical information.

Two independent cohorts were considered because the original PROFUND study was conducted in hospitalized patients like in cohort H, but cohort A received an intervention involving a case management program with potential implications for prognosis. For the comparative analysis of the two cohorts, we used an independent-samples *t*-test for continuous variables and Pearson’s chi-square test for categorical variables.

The discriminatory properties of the model were evaluated by estimating Kaplan–Meier survival curves for the four PROFUND risk groups. Survival distributions were compared using the log-rank (Mantel–Cox) test as the primary method. The Breslow (generalized Wilcoxon) and Tarone–Ware tests were additionally performed as sensitivity analyses to account for potential differences in early- and intermediate-event weighting. We assessed discrimination for 12-month mortality using receiver operating characteristic (ROC) curve analysis and quantified it by the AUC with 95% CI. The vital status at 12 months was completed for all participants, permitting a fixed-horizon ROC/AUC analysis (death ≤ 12 months vs. survival > 12 months). We compared AUCs obtained from the two independent cohorts using an approximate z-test on their standard errors.

Calibration was first evaluated by comparing observed 12-month mortality within each PROFUND score group against the expected mortality reported for the original PROFUND validation cohort [13]. We summarized agreement using observed–expected differences and observed/expected ratios and displayed them using calibration plots. Given evidence of miscalibration, standard intercept-and-slope recalibration was performed separately within each cohort. For each patient, the expected probability of 12-month death was assigned according to their PROFUND stratum using the original validation-cohort risks and transformed into the logit scale. A logistic regression model was then fitted with 12-month death as the dependent variable and the logit of the original expected risk as the sole covariate, yielding a recalibrated intercept and slope.

Statistical analyses, calibration plots, and recalibration models were performed using IBM SPSS Statistics for Windows, version 28.0 (IBM Corp., Armonk, NY, USA).

The study was approved by the hospital ethics committee (reference No. 20250097).

## 3. Results

**Figure 1:**
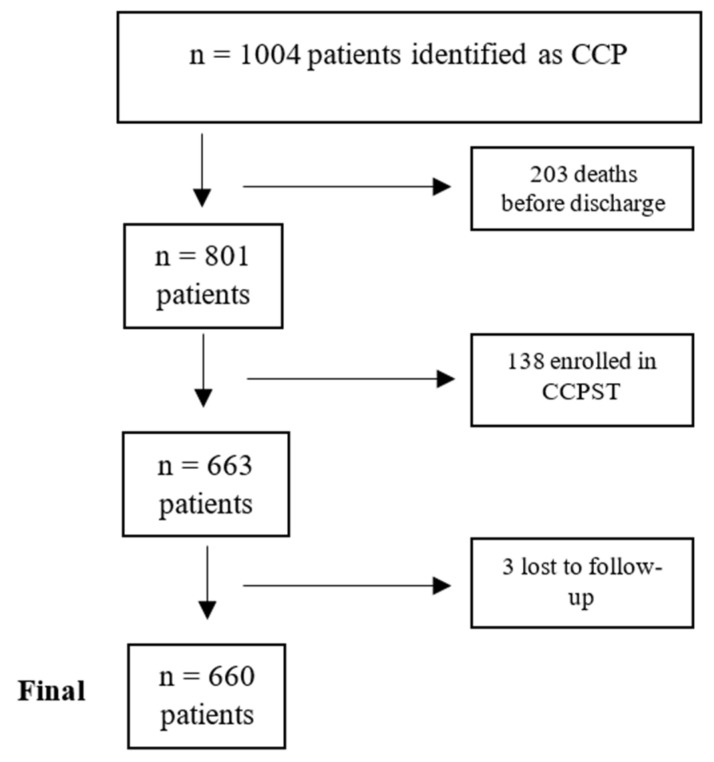
Identification of cohort H.

Cohort H included 660 patients, and cohort A included 540. Figure 1 shows that 1004 patients hospitalized with CCP criteria were initially identified. From this population, 203 were excluded for dying before discharge; 138 were excluded because they were already enrolled in the CCPST. Additionally, three patients were lost to follow-up, as it was not possible to determine whether they had died, further supporting the completeness and robustness of outcome ascertainment. During the same period, 4217 patients were admitted to the Medicine Ward, of whom 534 died before discharge (mortality rate of 12.7%). CCP accounted for 21.7% of the patients who were discharged and 38% of those who died.

Table 3 presents the sociodemographic and clinical characteristics of the two cohorts. Cohort A was slightly older (82.8 ± 8.0 vs. 81.3 ± 8.9 years) and included a higher proportion of females (54.8% vs. 49.7%). In this population, there was a higher proportion of patients without a caregiver (19.4% vs. 9.8%), and when a caregiver was present, children were more frequently the primary caregivers (36.3% vs. 26.2%). In cohort H, “other” caregivers were more prominent, corresponding predominantly to patients residing in long-term care facilities (14.8% vs. 3.1%). The results regarding cohabitants followed the same pattern observed for caregivers, with a higher proportion of patients in A living alone (15.7% vs. 11.2%), whereas patients in H more frequently resided in long-term care facilities (14.7% vs. 3.5%). Regarding healthcare utilization, cohort A had more emergency department visits in the previous year (4.75 ± 4.6 vs. 3.55 ± 3.0), a similar number of hospital admissions (1.84 ± 1.65 vs. 1.81 ± 1.0), and fewer inpatient days (19.7 ± 23.3 vs. 25.9 ± 32). At 12 months, mortality was lower in A (30.2% vs. 38.0%), and the mean PROFUND score was marginally lower (7.7 ± 5.0 vs. 8.3 ± 5.3). The risk distribution was also more favorable in A: high risk 27.8% vs. 34.8%; intermediate–high 23.5% vs. 21.1%; intermediate–low 32.0% vs. 30.5%; and low 16.7% vs. 13.6%.

**Figure 2:**
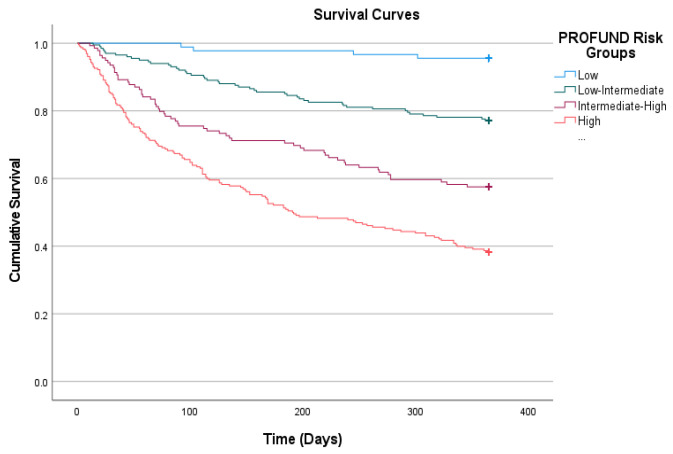
Kaplan–Meier curve for annual mortality in cohort H.

**Figure 3:**
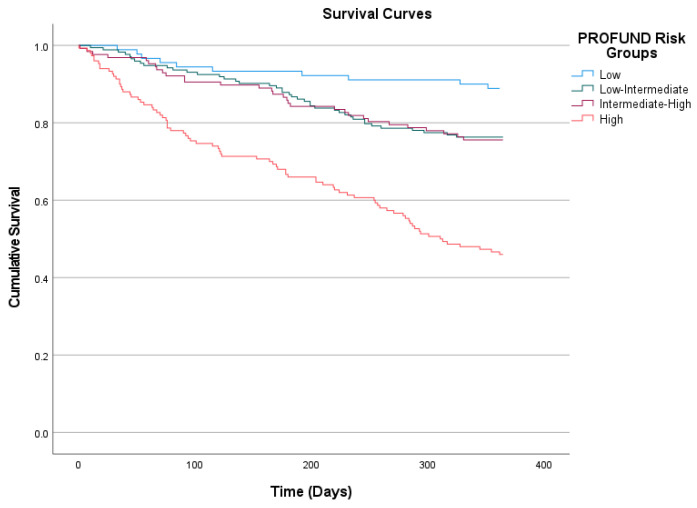
Kaplan–Meier curve for annual mortality in cohort A.

To assess discrimination, patients were stratified into the risk groups defined by the PROFUND scale, and Kaplan–Meier survival curves were generated for each group. The curves showed clear separation across the different groups in cohort H (Figure 2), although the distinction was less marked in cohort A (Figure 3), particularly among the intermediate-risk groups. Significant differences (*p* < 0.001) in survival were observed between PROFUND risk groups in both populations using the log-rank (126.341 in H, 67.832 in A), Breslow (125.959 in H, 66.528 in A), and Tarone–Ware tests (126.657 in H, 67.305 in A), indicating consistent separation of survival curves throughout follow-up.

**Figure 4:**
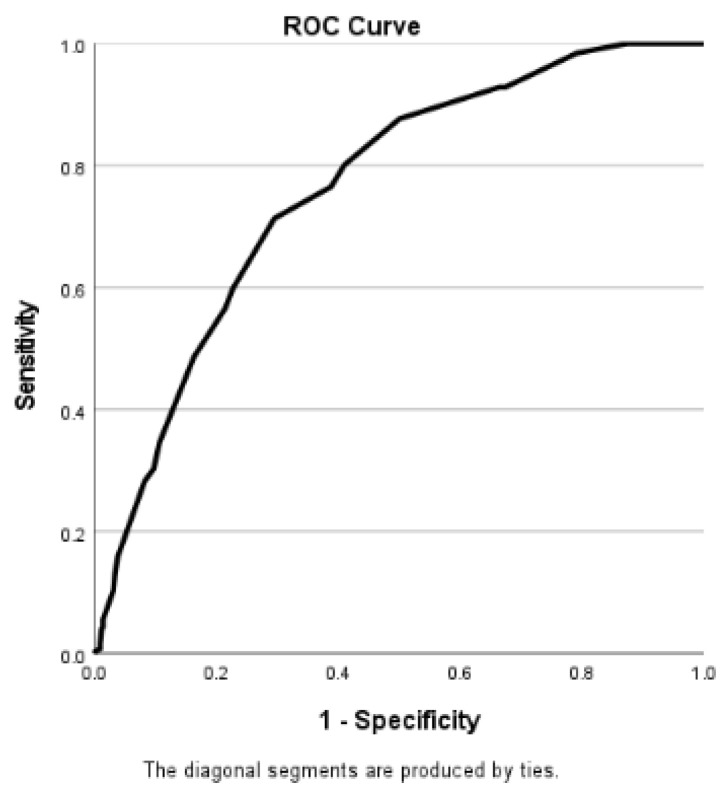
ROC curve for cohort H.

In cohort H, the AUC for 12 months of mortality was 0.760 (95% CI 0.724–0.797; *p* < 0.001), indicating good discriminatory performance of the PROFUND score (Figure 4).

**Figure 5:**
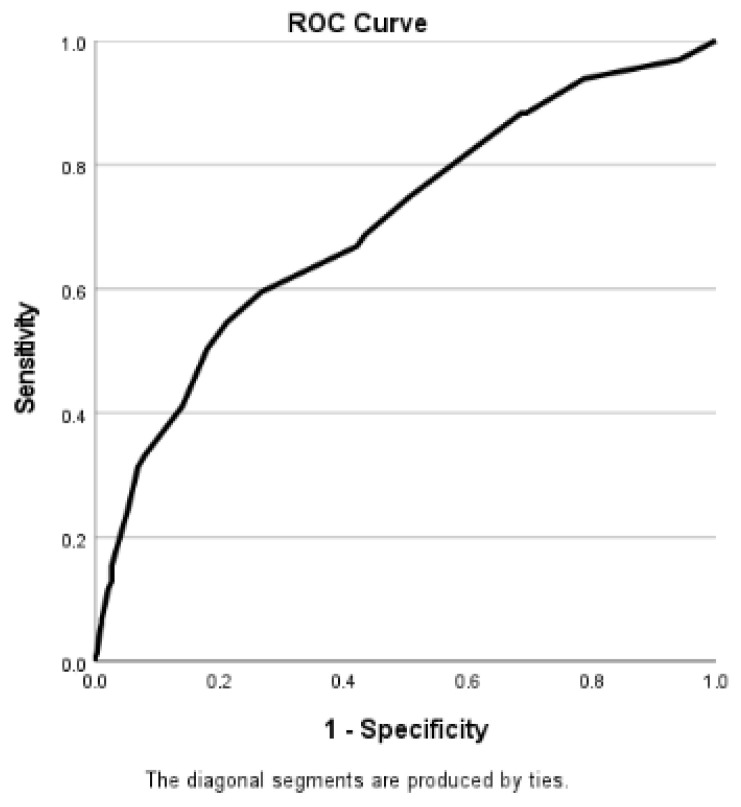
ROC curve for cohort A.

In cohort A, the AUC for 12 months of mortality was 0.705 (95% CI 0.656–0.754; *p* < 0.001), indicating moderate-to-good discriminatory performance of the PROFUND score (Figure 5).

The difference between AUCs, assuming independent samples, was 0.055, and an approximate z-test (z = 1.75; *p* = 0.08) did not indicate a statistically significant difference between cohorts.

**Figure 6:**
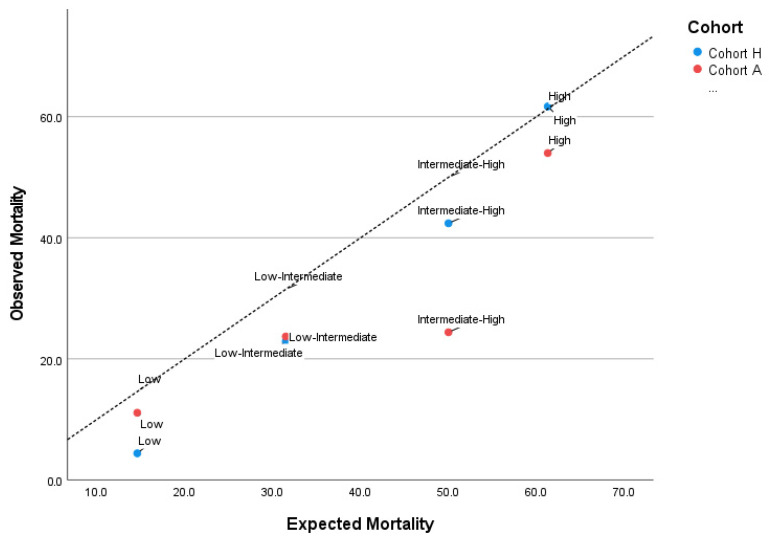
Observed mortality by expected mortality by cohort. Points below the diagonal line indicate that the original PROFUND model systematically overestimates mortality risk in the study population.

Calibration by PROFUND risk strata in cohort H (Figure 6) showed agreement in the high-risk group (61.7% vs. 61.3%; O/E = 1.01), but observed mortality was lower than expected in the other strata (O/E ranging from 0.30 to 0.85), indicating systematic overestimation of mortality in lower-risk patients. In cohort A, the PROFUND index consistently overestimated mortality, particularly in the intermediate–high group (24.4% vs. 50%; O/E = 0.49), resulting in a pessimistic calibration across strata. The diagonal dashed line indicates perfect calibration based on the original PROFUND validation cohort.

Intercept and slope recalibration were performed separately within each cohort because calibration differed between settings. In cohort H, the recalibration intercept was β0 = −0.204 and slope β1 = 1.443 (*p* < 0.001 for slope), and in cohort A, β0 = −0.555 and β1 = 0.942 (*p* < 0.001 for slope). Table 4 presents recalibrated predicted risks (mean $p_{\mathrm{recal}}$ by stratum) for Cohort H: 6.0% (low risk), 21.0% (low–intermediate risk), 44.9% (intermediate–high risk), and 61.3% (high risk); and for Cohort A: 9.8% (low risk), 21.6% (low–intermediate risk), 36.5% (intermediate–high risk), and 47.0% (high risk). These recalibrated probabilities aligned the overall predicted risk with the observed 12-month mortality within each cohort (38.0% in Cohort H and 30.2% in Cohort A).

## 4. Discussion

In this real-world retrospective study, the PROFUND index demonstrated clinically useful discrimination for 12-month mortality among CCPs across both inpatient and ambulatory care settings. Nevertheless, calibration analyses indicated an overestimation of mortality, especially in cohort A and among patients in the intermediate–high risk strata. These results suggest that while the PROFUND index remains useful for mortality risk stratification, its absolute risk estimates are not directly transferable to integrated case management settings and require recalibration.

Overall, patients‘ characteristics and outcomes reflect a typical real-world population of CCPs managed in routine clinical settings. This CCP population shares common characteristics with polypathological patients, in whom the PROFUND index has been applied in Spain, namely, a mean age above 75 years, multimorbidity, polypharmacy, and greater utilization of healthcare services.

The prevalence of 23.8% of CCPs among all patients admitted to an Internal Medicine department highlights the importance of this subgroup, with their proportionally greater contribution to overall mortality rates being one of the reasons for applying an appropriate prognostic scale to this population. Comparisons with other studies are hindered by the differing definitions of complexity, with prevalence ranging from 71% when a simple numerical count of chronic conditions is used to 14.7% when only the most complex cases are considered [21].

Overall, the two cohorts were statistically different across most sociodemographic, clinical and healthcare-utilization variables, despite exhibiting broadly comparable profiles of multimorbidity and chronic care needs. Differences were statistically significant for most variables, including age (*p* = 0.002), presence of a caregiver (*p* < 0.001), number of medications (*p* < 0.001), emergency department visits (*p* < 0.001), days of hospitalization (*p* < 0.001), number of chronic diseases (*p* < 0.001), and one-year mortality (*p* = 0.004). Despite these contrasts, both populations shared similar multimorbidity burdens, comparable distributions of chronic conditions, and overlapping PROFUND risk profiles, supporting their conceptual alignment as CCP populations while reinforcing the need to analyze them as distinct cohorts. Differences in caregiver structure between cohorts may also have influenced prognostic performance. Cohort A included a higher proportion of patients living alone or without a formal caregiver, which may partly reflect greater functional reserve and the ability to remain in the community despite multimorbidity. Conversely, cohort H included a larger proportion of institutionalized patients and formal caregiving arrangements, potentially reflecting more advanced frailty and dependency. However, socially vulnerable patients living alone may also represent a subgroup particularly responsive to integrated case management interventions and partially compensate for limitations in informal support networks. In this context, caregiver-related variables included in the original PROFUND model may carry different prognostic implications across inpatient and ambulatory integrated care settings, contributing to calibration drift despite preserved discriminatory performance.

The 12-month Kaplan–Meier survival curves demonstrated good discriminatory capacity between groups in both cohorts. The concordant significance across the log-rank, Breslow, and Tarone–Ware tests supports the robustness of PROFUND’s discriminatory performance, suggesting that survival differences are not restricted to either early or late follow-up periods.

The PROFUND index adequately discriminated the 12-month mortality risk in CCPs in both inpatient and ambulatory settings, with numerically higher performance among hospitalized patients, although without statistically significant differences. The implementation of a case management program for ambulatory CCPs and its impact on prognosis may contribute to this trend and should be further explored in future research.

Table 5 compares the AUC values from the study populations with those from cohorts of polypathological patients in previous studies where the PROFUND index was applied. In our study, the PROFUND score showed good discrimination in the hospital cohort (AUC 0.760; 95% CI 0.724–0.797) and moderate discrimination in the ambulatory cohort (AUC 0.705; 95% CI 0.656–0.754). These estimates are comparable to the derivation and validation cohorts from the original study reported by Bernabeu-Wittel et al. [13] (AUC 0.77 and 0.70, respectively) and the external validation in Internal Medicine by Díez-Manglano et al. [14] (AUC 0.725; 95% CI 0.670–0.781), supporting the transportability of the PROFUND index into our context. Our findings align with both the original derivation and external validations, indicating sustained 12-month discriminatory performance in CCPs. This may partly reflect similarities between the Portuguese and Spanish healthcare systems, particularly regarding universal public healthcare coverage, aging populations, and the organizational role of Internal Medicine in managing multimorbid patients.

However, external calibration against the original PROFUND validation cohort showed that absolute risk estimates were not fully transportable to the study setting. In both cohorts, the original PROFUND risks tended to overestimate observed mortality, most notably in the ambulatory cohort and particularly in the intermediate–high stratum. In contrast, calibration in the highest-risk stratum was close in Cohort H, suggesting that PROFUND may be particularly reliable for identifying very high-risk discharged inpatients.

This study supports the use of the PROFUND index for risk stratification in CCPs across settings, but absolute risk should ideally be adapted to the local baseline risk. We therefore performed standard intercept and slope recalibration separately in each cohort. The recalibrated parameters provided a practical method to update expected 12-month mortality probabilities while preserving the original PROFUND structure and risk group definitions. For routine clinical use, recalibration can be implemented as updated risk estimates per PROFUND stratum for hospitalized and ambulatory settings.

The systematic overestimation of mortality observed in the ambulatory cohort may reflect differences between the integrated case management model and the original PROFUND derivation population. Patients included in the CCPST were followed through a multidisciplinary case management model with home-based support, longitudinal monitoring, individualized care planning, and closer coordination between healthcare professionals. These interventions may have modified the baseline mortality risk without substantially altering relative risk stratification, which could explain the preservation of discrimination alongside deterioration in calibration. In this context, PROFUND appears to retain value as a risk-stratification tool, while absolute mortality estimates become less transportable without local recalibration. This pattern suggests that recalibration may be necessary when prognostic tools developed in conventional inpatient populations are applied to integrated longitudinal care models.

The observed differences in mortality in the intermediate–high risk strata between cohort A and the original cohort raise the hypothesis that these patients may particularly benefit from integrated case management strategies, although this interpretation requires confirmation in prospective comparative studies. These individuals may have sufficient baseline vulnerability to benefit from intensified multidisciplinary follow-up while still maintaining a degree of physiological reserve that allows potentially avoidable decompensations, hospitalizations, and treatment fragmentation to be mitigated through coordinated care. Conversely, lower-risk patients may initially benefit from less resource-intensive follow-up strategies, whereas extremely high-risk patients may have mortality trajectories that are less modifiable, despite intensive intervention. This further supports the role of the PROFUND index in optimizing resource allocation.

Although these interpretations cannot be confirmed within the retrospective observational design of the present study, our findings support the potential utility of prognostic tools for mortality prediction and for guiding the tailored intensity of integrated care interventions. In this framework, the PROFUND index may help identify patients more likely to benefit from different levels of case management intensity, thereby contributing to a more efficient and individualized organization of complex chronic care pathways.

The rationale and evidence [22,23] indicate that case management programs require time for case managers to implement effective interventions. From a clinical perspective, the ability of the PROFUND index to stratify short-term mortality risk may support individualized care planning, prioritization of follow-up intensity, and timely referral to palliative care or advanced care planning pathways in CCPs. The PROFUND index showed a better performance compared to the intuition of the clinicians to predict death during the first year of follow-up after hospitalization [4], and better or the same performance compared with NECPAL ICO-CCOMS© (Palliative Needs Assessment Tool developed by the World Health Organization Collaborating Center—Catalan Institute of Oncology) in a time frame from 3 to 24 months [24]. The use of the PROFUND index enhances clinical accuracy by directing resources toward those with the greatest potential for benefit, and optimizes resources use by enabling better continuity of care planning, thereby avoiding futile interventions.

The main limitation of this study was the retrospective design and reliance on EHR documentation. In addition, recalibration parameters were estimated within the same datasets in which calibration was assessed; therefore, recalibrated performance represents clear (in-sample) calibration and may require confirmation in additional CCP cohorts. Another limitation was that the ambulatory cohort was exposed to a multidisciplinary case management program and no comparable non-exposed ambulatory cohort was available; so, the study does not allow causal inference regarding the effect of the intervention on mortality outcomes.

One additional limitation relates to the inclusion of cohorts recruited during different time periods, which may have introduced temporal bias related to changes in the organization and management of CCPs over time. Consequently, some observed differences between cohorts may partly reflect secular trends in healthcare delivery and chronic disease management rather than differences exclusively attributable to the care setting. Although the study was not designed to estimate causal intervention effects, residual confounding cannot be excluded, and comparisons between cohorts should therefore be interpreted cautiously.

Despite these limitations, the study reflects routine clinical practice and provides clinically relevant information on the use of the PROFUND index in contemporary complex chronic care. Health systems implementing integrated care models for CCPs may benefit from incorporating recalibrated prognostic tools into EHR platforms to support individualized care planning and more accurate mortality risk stratification.

## 5. Conclusions

The PROFUND index is a useful prognostic tool for CCPs across care settings but requires local recalibration to ensure accurate risk estimation, particularly in ambulatory populations integrated in case management programs.

## Figures and Tables

**Table 1:** PROFUND Index [13].

Variable	Points
Age ≥ 85 years	3
Active neoplasia	6
Dementia	3
III-IV NYHA dyspnea or 3–4 mMRC	3
Delirium during the last hospital admission	3
Hb < 10 g/dL	3
Barthel index < 60	4
Absence of caregiver or caregiver other than spouse	2
≥4 hospital admissions over the last 12 months	3

NYHA: New York Heart Association; mMRC: Modified Medical Research Council; Hb: Hemoglobin.

**Table 2:** Death risk quartiles.

Groups	Points	Mortality [13] %
Low risk	0–2	14.6
Low–intermediate risk	3–6	31.5
Intermediate–high risk	7–10	50
High risk	≥11	61.3

**Table 3:** Characterization of the cohorts.

Characteristic	Cohort H	Cohort A		*p*
Number (*n*)	660	540		
Age in years, mean (SD)	81.3 (8.9)	82.8 (8)	t(1198) = −3.030	0.002
Female sex, *n* (%)	328 (49.7)	296 (54.8)	X^2^ = 3.117	0.077
Marital status, *n* (%)				
- Married/Common-law	362 (54.8)	266 (49.3)	X^2^ = 6.667	0.083
- Single	27 (4.1)	17 (3.1)		
- Divorced	27 (4.1)	32 (5.9)		
- Widowed	240 (36.4)	225 (41.7)		
- Unknown	4 (0.6)	0 (0)		
Caregiver relationship, *n* (%)				
- No caregiver	65 (9.8)	105 (19.4)	X^2^ = 92.937	<0.001
- Spouse	268 (40.6)	150 (27.8)		
- Daughter/Son	173 (26.2)	196 (36.3)		
- Other family member	33 (5)	48 (8.9)		
- Informal caregiver	23 (3.5)	24 (4.4)		
- Other	98 (14.8)	17 (3.1)		
Cohabitants, *n* (%)				
- None	74 (11.2)	85 (15.7)	X^2^ = 60.437	<0.001
- Spouse	245 (37.1)	192 (35.6)		
- Daughter/Son	136 (20.6)	129 (23.9)		
- Spouse and sons	65 (9.8)	59 (10.9)		
- Other family member	26 (3.9)	49 (9.1)		
- Informal caregiver	12 (1.8)	6 (1.1)		
- Parents	5 (0.8)	1 (0.2)		
- Other	97 (14.7)	19 (3.5)		
Number of drugs, mean (SD)	9.85 (3.1)	10.7 (3)	t(1198) = −4.681	<0.001
Emergency episodes, mean (SD)	3.55 (3)	4.75 (4.6)	t(903) = −5.147	<0.001
Number of hospitalizations, mean (SD)	1.81 (1)	1.84 (1.65)	t(877) = −0.300	0.754
Days of hospitalization, mean (SD)	25.9 (32)	19.7 (23.3)	t(1198) = 3.624	<0.001
Chronic diseases, mean (SD)	3 (0.8)	3.2 (0.8)	t(1155) = −4.047	<0.001
One-year mortality, *n* (%)	251 (38)	163 (30.2)	X^2^ = 8.089	0.004
PROFUND index, mean (SD)	8.3 (5.3)	7.7 (5)	t(1198) = 2.088	0.037
Risk PROFUND group *n* (%)				
- Low	90 (13.6)	90 (16.7)		
- Low–intermediate	201 (30.5)	173 (32)		
- Intermediate–high	139 (21.1)	127 (23.5)		
- High	230 (34.8)	150 (27.8)		

**Table 4:** Calibration by PROFUND risk strata: original expected vs. observed vs. recalibrated.

PROFUND Stratum	Expected (%) (Original)	Cohort H *n*	Cohort H Deaths	Cohort H Observed (%)	Cohort H Recalibrated (%)	Cohort A *n*	Cohort A Deaths	Cohort A Observed (%)	Cohort A Recalibrated (%)
Low (0–2)	14.6	90	4	4.4	6.0	90	10	11.1	9.8
Low–Intermediate (3–6)	31.5	201	46	22.9	21.0	173	41	23.7	21.6
Intermediate–High (7–10)	50.0	139	59	42.4	44.9	127	31	24.4	36.5
High (≥11)	61.3	230	142	61.7	61.3	150	81	54.0	47.0

**Table 5:** Comparative AUC table.

Study/Setting	Time (Months)	Population	*n*	AUC (95% CI)
Bernabeu-Wittel et al. (derivation cohort) [13]	12	Polypathological patients in internal medicine and geriatric areas	757	0.77 (0.731–0.805)
Bernabeu-Wittel et al. (validation cohort) [13]	12	Polypathological patients in internal medicine and geriatric areas	768	0.70 (0.67–0.74)
Bernabeu-Wittel et al. [8]	12	Polypathological patients in internal medicine and geriatric areas	1070	0.67 (0.63–0.69)
Díez-Manglano et al. [14]	12	Polypathological patients in internal medicine area	333	0.725 (0.670–0.781)
Díez-Manglano et al. [14]	12	Polypathological patients in geriatric areas	132	0.546 (0.448–0.644)
Moretti et al. [15]	12	Inpatients (comparative)	92	0.712 (0.607–0.817)
Cohort H	12	Hospitalized Complex Chronic Patients	660	0.760 (0.724–0.797)
Cohort A	12	Ambulatory Complex Chronic Patients	540	0.705 (0.656–0.754)
Pilar Bohórquez Colombo et al. [19]	24	Polypathological patients in ambulatory	446	0.622 (0.556–0.689)

## Data Availability

The datasets used in the current study are available from the corresponding author on reasonable request.

## Abbreviations

CCP	Complex Chronic Patient
AUC	Area Under the Curve
CCPST	Complex Chronic Patients Support Team
EHR	Electronic Health Record
ROC	Receiver Operating Characteristic
CI	Confidence Interval
NYHA	New York Heart Association
mMRC	Modified Medical Research Council
Hb	Hemoglobin
SPSS	Statistical Package for the Social Sciences
